# Psychometric properties of the Chinese version of the Posttraumatic Growth Inventory-Short Form among Chinese-speaking cancer patients

**DOI:** 10.3389/fpsyg.2026.1838732

**Published:** 2026-09-23

**Authors:** Yuanyuan Hu, Hao Yang, Jingjing Wang, Rui Liu, Xiaochun Wang, Yinghua Ji, Qingqin Zhang, Liuyang Zhao, Yi Jing, Yuepu Gao, Zheng Zhang, Nizuwan Azman, Lifang Zhang, Mohammad Farris Iman Leong Bin Abdullah, Mohd Salami Ibrahim

**Affiliations:** 1 First Affiliated Hospital of Henan Medical University, Xinxiang, Henan, China; 2 Research, Innovation and Networking Unit, Tun Abdullah Ahmad Badawi Cancer Centre, Universiti Sains Malaysia, Kepala Batas, Pulau Pinang, Malaysia; 3 Department of Psychiatry and Mental Health, Faculty of Medicine, Universiti Sultan Zainal Abidin, Kuala Terengganu, Terengganu, Malaysia; 4 Department of Health Professional Education, Faculty of Medicine, Universiti Sultan Zainal Abidin, Kuala Terengganu, Terengganu, Malaysia

**Keywords:** Posttraumatic Growth Inventory-Short Form, cancer, reliability, validity, China

## Abstract

**Background:**

This study translated the English version of the Posttraumatic Growth Inventory-Short Form (PTGI-SF) into Chinese and evaluated the psychometric properties of the Chinese version of the PTGI-SF.

**Methods:**

This study was divided into two phases. Phase I translated the English version of the PTGI-SF into Chinese and evaluated its content and face validity. In Phase II, the study participants were administered the Chinese versions of the PTGI-SF, the Benefit Finding Scale (BFS) as comparators to assess convergent validity, and the Hospital Anxiety and Depression Scale (HADS) as a comparator to evaluate discriminant validity. The factor structure of the Chinese version of the PTGI-SF was examined using exploratory (EFA) and confirmatory factor analysis (CFA), while the reliability of the Chinese version of the PTGI-SF was determined by measuring internal consistency and test–retest reliability.

**Results:**

A total of 514 cancer patients with mixed cancer types completed the study. The Chinese version of the PTGI-SF exhibited good content and face validity. The internal consistency of the Chinese version of the PTGI-SF was good, with a Cronbach’s α of 0.890 and a McDonald’s omega of 0.884, while the test–retest reliability was also acceptable, with an intraclass correlation coefficient of 0.785 (*p* < 0.001). Convergent validity of the Chinese version of the PTGI-SF was supported, as Pearson’s correlation coefficients between the total BFS score and both the PTGI-SF total score and several subscale domains were greater than 0.5. The discriminant validity of the instrument was also achieved, as Pearson’s correlation coefficients between the total HADS score and both the PTGI-SF total score and several subscale domains were less than 0.3. EFA and CFA confirmed that the Chinese version of the PTGI-SF consisted of only one factor.

**Conclusion:**

The Chinese version of the PTGI-SF had good psychometric properties and is ready for use in the Chinese-speaking cancer population.

## 1 Introduction

Cancer is one of the major non-communicable diseases that contribute to a significant disease burden and mortality globally. Cancer rates are increasing, rising from a total of 10.1 million cases and 6.1 million deaths in 2000 to a total of 20 million cases and 10 million deaths in 2022 worldwide (World Health Organization, 2026). Cancer is expected to project a continuously increasing trend toward 2050, in which the number of cancer cases may rise to 35.3 million and contribute to 18.5 million deaths (World Health Organization, 2026). In China, the number of new cancer cases reached 4.8 million, and cancer contributed to 2.57 million deaths (Diao et al., 2025). Cancer contributes not only to mortality, but it also leads to a diminished quality of life among cancer patients, attributed to poor physical well-being and mental well-being (Firkins et al., 2020).

Despite the negative impact of cancer on the lives of cancer survivors, there are also a number of positive impacts. One of the positive psychological traits that follows the occurrence of cancer is posttraumatic growth (PTG), which is a transformational psychological phenomenon characterized by improved psychological functioning beyond that of the state prior to the diagnosis of cancer, resulting from the extreme stress or trauma of living with cancer and the adverse effects of its treatment. PTG comprises the summation effects of its five components, namely better appreciation of life, greater new possibilities in life, improved spiritual development, greater personal strength, and a greater degree of relating to others (Leong Abdullah et al., 2019; Nik Jaafar et al., 2022). PTG is reported to develop following diagnosis and treatment of cancer, and it may follow an increasing trend over time up to 18 to 24 months after cancer treatment (Harding, 2018). PTG is reported to increase the quality of life of cancer patients and, essentially, is an important psychological outcome to be targeted by psychosocial interventions (Zhang et al., 2026).

In the context of the popularity of PTG-related research, a bibliographic analysis of research articles from the Web of Science Core Collection and CiteSpace, which analyzed a total of 2,596 PTG-related references from 1996 to 2020, revealed that more and more researchers are now paying attention to PTG and cancer survivors. Common research topics related to PTG were influencing factors of PTG, coping and intervention measures, and the research objects. The emerging trend of research in the field of PTG was represented by keywords, such as “adjustment,” “oncology,” “stage breast cancer,” and “perceived benefit.” Several interventions have been reported to improve PTG, including psychosocial interventions, supportive-expressive group therapy, mindfulness-based interventions, and behavioral therapy. In terms of the study subjects, cancer survivors and accident and natural disaster victims were most extensively studied. However, PTG-related studies in patients with chronic diseases (stroke, coronary heart disease, hemodialysis, and diabetes mellitus) are also exhibiting an increasing trend (Kou et al., 2021). Regarding the evolution of instruments used to measure PTG, after the development of the PTG Inventory (PTGI), several other related instruments emerged, such as the PTG Inventory-Short Form (PTGI-SF), the PTG Inventory-Extended (PTGI-X), and the Physical PTG Inventory (P-PTGI).

The PTGI is the original 21-item, five-domain instrument used to measure PTG (Tedeschi and Calhoun, 1996). The PTGI-X is the latest version of the PTGI; it consists of 25 items with four additional items on spiritual-existential change to further strengthen the assessment of PTG (Tedeschi et al., 2017). The PTGI-SF is a shorter version derived from the 21-item PTGI and consists of 10 items designated to five domains. Of all the tools that measure PTG, it is the most suitable tool to assess PTG among cancer patients. The use of PTGI-SF in cancer patients is so beneficial because of its much shorter duration of administration (it consists of only 10 items), which allows cancer patients with physical discomfort and short concentration and attention to complete it faster and thus minimize response bias. Moreover, the PTGI-SF can also substitute for the PTGI without any loss of information (Cann et al., 2010).

In essence, the PTGI-SF is a suitable tool for assessing changes in the degree of PTG over time in China and regions where Chinese is widely spoken. The literature search revealed that there is a Chinese version of the PTGI and its short form (the latter developed from the validated PTGI) was validated for use among Chinese Shidu parents but not for Chinese-speaking cancer patients (Xu et al., 2023). To the best of our knowledge, to date, PTGI-SF has not been translated into Chinese or adapted for use in the Chinese-speaking cancer population, such as those from China, Taiwan, Hong Kong, and Macao. Hence, this study aimed to translate the original English version of the PTGI-SF into Chinese and evaluate the internal consistency and test–retest reliability, face, content, convergent, discriminant validity, exploratory factor analysis, and confirmatory factor analysis of the Chinese version of the PTGI-SF.

## 2 Materials and methods

### 2.1 Phase I

#### 2.1.1 Translation and back-translation of the Posttraumatic Growth Inventory-Short Form into Chinese

The original English version of the PTG Inventory-Short Form (PTGI-SF) was forward-translated into Chinese by two bilingual, native Chinese-speaking language experts from Henan Medical University. Then, the Chinese draft of the PTGI-SF was back-translated by a native English bilingual language expert who had not seen the original English version of the PTGI-SF before. Then, the three language experts and the principal investigator harmonized the translated and back-translated copies of the PTGI-SF before these copies were passed to a group of content experts for further improvement and assessment of content validity.

#### 2.1.2 Assessment of content validity of the Posttraumatic Growth Inventory-Short Form by an expert panel

A content expert panel was formed, which consisted of six experts: two psychiatrists, a clinical psychologist, a psychologist, and two oncologists from Henan Medical University (formerly Xinxiang Medical University). The inclusion criteria for selection onto the expert panel were [1] at least 5 years of clinical experience in the field of mental health or oncology, [2] at least 5 years of research experience in the field of mental health or oncology, and [3] at least a PhD or MD graduate. The details of each of the experts are summarized in Supplementary material 1.

For assessment of content validity, the experts were instructed to evaluate the appropriateness of all items in the translated and back-translated versions of the PTGI-SF according to their allocation to each domain of the instrument in terms of clarity of semantic quality and syntax, sentence structure, relevance to the construct they measure, comprehensiveness, conceptual equivalence, redundancy, and cultural appropriateness. A scoring system was used for the assessment of each item: 1 = non-appropriate, 2 = somewhat appropriate, 3 = appropriate, and 4 = extremely appropriate. Scores of 1 and 2 were given a content validity item score of 0, and scores of 3 and 4 were given a content validity score of 1. The item-level content validity index (I-CVI) was calculated as the number of experts rating the item as content valid (a score of 3 or 4) divided by the total number of experts. A score of 0.83 was considered acceptable for each item (Yusoff, 2019a). A universal agreement (UA) of 1 was scored for an item if all the experts agreed to give a content validity score of 1 to the item (a score of 3 or 4 in the assessment of the appropriateness of the item). Then, the content validity index of the scale based on the average method (S-CVI/Ave) was calculated by dividing the sum of all the I-CVI scores by the sum of all the items in the scale, whereby a score of 0.9 was considered acceptable (Yusoff, 2019a). As for the content validity index of the scale based on the UA method (S-CVI/UA), it was calculated by dividing the sum of the UA scores of the scale by the total number of items in the scale, wherein a score of 0.8 was considered adequate (Yusoff, 2019a).

#### 2.1.3 Assessment of the face validity of the Chinese version of the Posttraumatic Growth Inventory-Short Form in a pilot study

To assess the face validity of the Chinese version of the PTGI-SF, a pilot study was conducted with 30 native Chinese-speaking cancer patients undergoing treatment at the First Affiliated Hospital of Henan Medical University across diverse cancer types and stages. They were sampled using simple random sampling, whereby all the cancer patients treated in the First Affiliated Hospital of Henan Medical University were initially given a random number. Then, a computer-generated sequence of random numbers was used to select the participants until a total of 30 participants was reached.

The cancer patients were instructed to evaluate each of the items in the Chinese version of the PTGI-SF according to their allocation to each domain of the instrument in terms of clarity of semantic quality and syntax, sentence structure, relevance according to the construct which they measure, comprehensiveness, conceptual equivalence, cultural appropriateness, redundancy, and duration of administration. A scoring system was used for the assessment of each item: 1 = non-appropriate, 2 = somewhat appropriate, 3 = appropriate, and 4 = extremely appropriate. The scores of 1 and 2 were given a content validity item score of 0, and the scores of 3 and 4 were given a content validity score of 1. For the face validity index of the item (I-FVI), it was calculated as the sum of the scores of all the participants divided by the total number of participants. A score of 0.83 was considered acceptable for each item (Yusoff, 2019b). Universal agreement (UA) of 1 was scored for an item if all the participants agreed to give a face validity score of 1 to the item (a score of 3 or 4 in the assessment of appropriateness of the item). Then, the face validity index of the scale based on the average method (F-CVI/Ave) was calculated by dividing the sum of all the I-FVI scores by the sum of all the items in the scale, whereby a score of 0.9 was considered acceptable (Yusoff, 2019b). As for the face validity index of the scale based on the UA method (S-CVI/UA), it was calculated by dividing the sum of the UA scores of the scale by the total number of items in the scale, wherein a score of 0.8 was considered adequate (Yusoff, 2019b).

### 2.2 Phase II

#### 2.2.1 Subject recruitment

This validation study was conducted from November 23, 2025 to February 1, 2026. This study was approved by the Human Research Ethics Committee of the Henan Medical University (code: EC-025-213). It was conducted according to the standard ethical practices established by the Declaration of Helsinki (1964) and its subsequent amendments.

The required sample size was calculated using an *a priori* sample size calculator for structural equation modeling (SEM) [citation/tool name], based on parameters for confirmatory factor analysis (CFA): an anticipated effect size of 0.2 (derived from the validation study of the original English PTGI-SF (Cann et al., 2010)), which was 0; type I error = 0.05, statistical power of 0.80, 10 observed variables, and 5 latent variables. Hence, the estimated sample size needed for this study was 400 subjects (inclusive of a 10% drop-out rate). Since exploratory factor analysis (EFA) was also performed and required another cohort of participants, the estimation of sample size was based on the cases-per-variable ratios. According to Bentler and Chou (1987), the cases-per-variable ratio should be at least fivefold the number of items in the scale. Dixon (2005) recommended that the sample size for exploratory factor analysis should be at least 10 times the number of items involved in the analysis. Hence, the rule of cases-per-variable ratio of at least 10 was applied, which yielded a minimum sample size required for EFA in this study of 110 (inclusive of a 10% drop-out rate). In summation, the estimated number of subjects required for this study was 510 subjects.

The source population in Phase II of this study comprised all cancer patients registered for treatment at the First Affiliated Hospital of Henan Medical University. The First Affiliated Hospital of Henan Medical University is a tertiary medical center for oncology referrals in Henan and neighboring provinces, such as Shanxi, Shaanxi, Anhui, Hebei, and Shandong. Hence, the hospital consists of approximately 2,500 to 3,000 cancer patients under its treatment and follow-up. Initially, posters advertising the study details for subject recruitment were pasted on the notice board of the Oncology Unit and the First Affiliated Hospital of Henan Medical University. Again, simple random sampling was applied, in which all cancer patients registered for treatment in the First Affiliated Hospital of Henan Medical University were identified and assigned a research number, excluding those who had already participated in the pilot study earlier. Then, a computer-generated random number sequence was used to select cancer patients. The selected patients were invited to visit the hospital for eligibility criteria screening. This process was repeated until the estimated sample size for the study was reached.

The inclusion criteria were as follows: (1) a histopathologically confirmed diagnosis of cancer of any type or stage, regardless of time elapsed since diagnosis; (2) age 18 years or older; and (3) literacy in Chinese (ability to read and write). The exclusion criteria included [1] those with physical disabilities that hindered their ability to answer questionnaires and [2] those with severe cognitive impairment that hindered their ability to answer questionnaires. Those who fulfilled all the eligibility criteria to participate in the study were invited to join the study. The purpose of the study, the study procedures, the risks and benefits of participating in the study, their rights to withdraw from the study with their data immediately discarded and not used in the study, and the assurance of anonymity of personal identity was explained to potential participants. All the potential participants signed written informed consent to participate in the study before they were enrolled.

#### 2.2.2 Data collection

All the participants were administered the demographic and clinical characteristics questionnaire, the Chinese versions of the PTGI-SF, the Benefit Finding Scale (BFS) as a comparative tool to assess convergent validity of the Chinese version of the PTGI-SF, and the Hospital Anxiety and Depression Scale (HADS) as a comparative tool to evaluate the discriminant validity of the Chinese version of the PTGI-SF. Then, 2 weeks after the first assessment, the Chinese version of the PTGI-SF was re-administered to 110 randomly selected participants from those who completed the first assessment to measure the test–retest reliability of the Chinese version of the PTGI-SF.

##### 2.2.2.1 Measures

(1) PTG Inventory-Short Form:

The PTGI-SF is a self-reported instrument used to measure the degree of PTG. It was derived from the original PTG, but it is shorter, with only 10 items designated to five domains (appreciation of life, relating to others, personal strength, spiritual growth, and new possibilities in life). Each item is scored on a Likert scale ranging from 0 to 5, and hence the total score range is from 0 to 50 (Cann et al., 2010).

(2) Benefit Finding Scale:

The BFS is a self-administered tool used to assess the degree of benefit finding. It consists of 22 items and six dimensions (acceptance, family relationship, personal growth, world outlook, social relationship, and healthy behavior). Each item is scored on a Likert scale ranging from 1 to 5, and the total score range is from 22 to 110. The higher the score, the greater the degree of benefit finding is (Weaver et al., 2008). The BFS was translated into Chinese and validated in the Chinese population (Liu et al., 2015).

(3) Hospital Anxiety and Depression Scale:

The HADS is a self-reported instrument used to assess the severity of depression and anxiety symptoms among people with medical illnesses, such as cancer. It consists of two subscales: the depression subscale, which is made up of seven items, and the anxiety subscale, which also consists of seven items. Each item is scored on a Likert scale ranging from 0 to 3. Hence, the total Depression subscale score is from 0 to 21, and the same applies to the Anxiety subscale score. The higher the score, the more severe the degree of depression or anxiety symptoms is. A score of 0 to 7 denotes normal, 8 to 10 indicates mild, and 11 to 21 indicates moderate to severe (Zigmond and Snaith, 1983). The HADS was translated into Chinese and validated in the Chinese cancer population (Li et al., 2016).

(4) Demographic and clinical characteristics:

The demographic characteristics documented in this study included age, gender, place of residence, and employment status. Age was self-reported by participants. The response options for gender were male or female; for place of residence the options were urban or rural; and for employment status the options were employed or unemployed.

The clinical characteristics collected in this study included types of cancer and mode of treatment received. The response options for types of cancer were breast cancer, colorectal cancer, lung cancer, or others; for mode of treatment received the options were one, two, three, or four or more modes of treatment.

#### 2.2.3 Statistical analysis

All the data were analyzed using the Statistical Package for Social Sciences version 29 (SPSS 29), except for CFA, which was computed using R version 4.3.1 (R Foundation for Statistical Computing, Vienna, Austria), accessed via the RStudio interface (version 2023.06.1, Build 524). Descriptive statistics were computed, with all the demographic and clinical characteristics presented in frequency and percentage, while the total PTGI-SF and its domain scores were reported as mean and standard deviation (SD).

As for the reliability assessment of the Chinese version of the PTGI-SF, the internal consistency of the total PTGI-SF score was computed, where a Cronbach’s α of 0.7 and confirmation by a McDonald’s omega of 0.7 were considered acceptable. For the test–retest reliability of the instrument, the intraclass correlation coefficient (ICC) between the baseline and the follow-up total and domain scores of the Chinese version of the PTGI-SF was computed. An ICC of 0.6 with *p* < 0.05 was considered acceptable.

For the measurement of convergent validity, the Pearson’s correlation coefficient between the domains and the total PTGI-SF and total BFS scores should be 0.5 with *p* < 0.05 to be considered acceptable (Chin and Yao, 2014). The higher the correlations, the greater the convergent validity. In terms of discriminant validity, the Pearson’s correlation coefficient between the domains and the total PTGI-SF and the HADS Depression and Anxiety subscale scores should be 0.3 or below to be considered acceptable (Henseler et al., 2015).

Factors were extracted using principal component analysis based on the Kaiser criterion Eigenvalue of at least 1. Data suitability for factor extraction was confirmed by a Kaiser–Meyer–Olkin (KMO) measure of sampling adequacy of at least 0.6 and a statistically significant Bartlett’s test of sphericity <0.05. Then, factor rotation should be performed using the designated factor to obtain a factor model in which all item loadings are highest on their designated factor and at least 0.4.

For the CFA, a polychoric correlation matrix was estimated given the ordinal structure of the scale, and subsequent models were compared using the robust diagonally weighted least squares mean- and variance-adjusted (WLSMV) estimator. Several factor models of the Chinese version of PTGI-SF should be evaluated, including the factor model suggested by EFA. In order to decide on the best-fitting factor model, several fit indices were compared between the factor models under comparison: [1] the chi-square to degree of freedom ratio (χ^2^/df), where a ratio of < 2 was considered excellent fit and a ratio of < 5 was considered acceptable good fit; [2] the comparative fit index (CFI), where a value of 0.95 was considered good fit; [3] the Tucker-Lewis index (TLI), where a value of 0.95 was considered good fit; [4] the goodness-of-fit index (GFI), where a value of 0.90 was considered good fit; and [5] the root mean square error of approximation (RMSEA), where a value of < 0.05 was considered good fit and < 0.08 was considered acceptable fit (Hu and Bentler, 1999).

#### 2.2.4 Item-level evaluation

Psychometric evaluation of the translated items was conducted using Rasch Partial Credit Model (PCM) analysis via the *eRm* package in R. The analysis assessed (1) threshold ordering to confirm the intended ordinal functioning of the 0–5 response categories; (2) item fit using Infit and Outfit mean square (MNSQ) statistics (acceptable range: 0.5–1.5); (3) targeting by comparing person ability estimates with item difficulty locations; (4) how effectively the scale could distinguish between individuals with different growth levels (person separation reliability); and (5) whether any items functioned differently for male versus female respondents. All analyses were performed on the full dataset of 400 participants with complete responses.

## 3 Results

### 3.1 Phase I

#### 3.1.1 Content validity of the Chinese version of the PTGI-SF

The content validity assessment for the Chinese version of the PTGI-SF is summarized in Table 1. The item-level content validity index (I-CVI) for all items ranged from 0.83 to 1.00, indicating excellent content validity. The S-CVI/Ave for the tool was 0.97, which denoted good S-CVI/Ave. The S-CVI/UA for this tool was 0.80, which denoted good S-CVI/UA.

**Table 1:** Content validity index of the Chinese version of the Posttraumatic Growth Inventory-Short Form.

Items	Expert 1	Expert 2	Expert 3	Expert 4	Expert 5	Expert 6	Expert in agreement	I-CVI	UA
Item 1	1	1	1	1	1	1	6	1	1
Item 2	1	1	1	1	1	1	6	1	1
Item 3	1	1	1	1	1	1	6	1	1
Item 4	1	1	0	1	1	1	5	0.83	0
Item 5	1	1	1	1	1	1	6	1	1
Item 6	1	1	1	1	1	1	6	1	1
Item 7	1	1	1	1	1	1	6	1	1
Item 8	1	1	1	1	1	1	6	1	1
Item 9	1	1	1	0	1	1	6	0.83	0
Item 10	1	1	1	1	1	1	6	1	1
Proportion relevance									
							S-CVI/Ave:	0.97	
							S-CVI/UA:		0.80

#### 3.1.2 Face validity of the Chinese version of the PTGI-SF

The face validity assessment for the Chinese version of the PTGI-SF is summarized in Table 2. The I-FVI of all items of the instrument ranged from 0.97 to 1.00, which indicated good FVI. The S-FVI/Ave for the tool was 0.99, which denoted good S-FVI/Ave. The S-FVI/UA for this tool was 0.80, which denoted acceptable S-FVI/UA.

**Table 2:** Face validity of the Chinese version of the Posttraumatic Growth Inventory-Short Form.

Items	1	2	3	4	5	6	7	8	9	10	11	12	13	14	15	16	17	18	19	20	21	22	23	24	25	26	27	28	29	30	Expert in agreement	I-FVI	UA
Item 1	1	1	1	1	1	1	1	1	1	1	1	1	1	1	1	1	1	1	1	1	1	1	1	1	1	1	1	1	1	1	30	1	1
Item 2	1	1	1	1	1	1	1	1	1	1	1	1	1	1	1	1	1	1	1	1	1	1	1	1	1	1	1	1	1	1	30	1	1
Item 3	1	1	1	1	1	1	1	1	1	1	1	1	1	1	1	1	1	1	1	1	1	1	1	1	1	1	1	1	1	1	30	1	1
Item 4	1	1	1	1	1	1	0	1	1	1	1	1	1	1	1	1	1	1	1	1	1	1	1	1	1	1	1	1	1	1	29	0.97	0
Item 5	1	1	1	1	1	1	1	1	1	1	1	1	1	1	1	1	1	1	1	1	1	1	1	1	1	1	1	1	1	1	30	1	1
Item 6	1	1	1	1	1	1	1	1	1	1	1	1	1	1	1	1	1	1	1	1	1	1	1	1	1	1	1	1	1	1	30	1	1
Item 7	1	1	1	1	1	1	1	1	1	1	1	1	1	1	1	1	1	1	1	1	1	1	1	1	1	1	1	1	1	1	30	1	1
Item 8	1	1	1	1	1	1	1	1	1	1	1	1	1	1	1	1	1	1	1	1	1	1	1	1	1	1	1	1	1	1	30	1	1
Item 9	1	1	1	1	1	1	1	1	1	1	1	0	1	1	1	1	1	1	1	1	1	1	1	1	1	1	1	1	1	1	29	0.97	0
Item 10	1	1	1	1	1	1	1	1	1	1	1	1	1	1	1	1	1	1	1	1	1	1	1	1	1	1	1	1	1	1	30	1	1
																															S-FVI/Ave	0.99	
																															S-FVI/UA		0.80

### 3.2 Phase II

#### 3.2.1 Participants and the total PTGI-SF scores

The demographic and clinical characteristics and the mean domain and total PTGI-SF scores of all the participants are summarized in Table 3. A total of 514 cancer participants completed this study with no missing data. In addition, 440 participants were involved in CFA, and another 74 participants were involved in EFA. Slightly more than half of the participants were male (*n* = 276, 53.7%), and half of the participants had lung, breast, or colorectal cancer (*n* = 260, 50.6%). The mean domain scores of the PTGI-SF ranged from 2.01 to 7.11, while the mean total PTGI-SF score was 25.15 (SD = 10.63).

**Table 3:** Socio-demographic and clinical characteristics and the domain and total PTGI-SF score of the participants.

Variables	Number of participants (*n*)	Percentage (%)
Age	62.12^#^	10.46^*^
Gender		
Male	276	53.7
Female	238	46.3
Place of residence		
Urban	148	28.8
Rural	366	71.2
Employment		
Unemployed	5	1.0
Employed	509	99.0
Types of cancer		
Breast cancer	56	10.9
Colorectal cancer	50	9.7
Lung cancer	154	30.0
Other	254	49.4
Mode of treatment received		
Single	79	15.4
Two	188	36.6
Three	144	28.0
Four or more	103	20.0
New possibilities	5.46^#^	2.38^*^
Spiritual development	2.01^#^	3.38^*^
Personal strength	4.95^#^	3.52^*^
Relationship with others	5.68^#^	3.22^*^
Appreciation of life	7.11^#^	3.11^*^
Total PTGI-SF	25.15^#^	10.63^*^

#, mean; *, standard deviation.

#### 3.2.2 Reliability of the Chinese version of the PTGI-SF

Assessment of the internal consistency of the Chinese version of the total PTGI-SF revealed that Cronbach’s α was 0.890, while McDonald’s omega was 0.884, which indicated that the Chinese version of the PTGI-SF had good internal consistency (Cronbach’s α > 0.7, McDonald’s omega > 0.7). Regarding assessment of test–retest reliability, the findings are presented in Table 4. The test–retest reliability of the domains of the PTGI-SF ranged from an ICC of 0.604 to 0.651, with all *p*-values < 0.001, whereas the test–retest reliability of the total PTGI-SF was an ICC of 0.785 (*p* < 0.001), which denoted a good test–retest reliability (ICC > 0.6).

**Table 4:** Test–retest reliability of the Chinese version of the Posttraumatic Growth Inventory-Short Form.

BIS items	Test–retest reliability (intraclass correlation coefficient)
Appreciation of life	0.634*
New possibilities	0.651*
Personal strength	0.604*
Spiritual development	0.633*
Relationship with others	0.616*
Total PTGI-SF	0.785*

*p < 0.001.

#### 3.2.3 Convergent validity of the Chinese version of the PTGI-SF

In the context of the convergent validity of the Chinese version of the PTGI-SF, the findings are summarized in Table 5. The Pearson’s correlation coefficient between the PTGI-SF domains and the total BFS ranged from 0.298 to 0.541, and all *p*-values were < 0.001, which indicated weak-to-moderate correlation coefficients. The Pearson’s correlation coefficient between the total PTGI-SF and the total BFS was 0.622 (*p* < 0.001), which indicated a moderate correlation coefficient with acceptable convergent validity (r > 0.5).

**Table 5:** Convergent validity of the Chinese version of the Posttraumatic Growth Inventory-Short Form.

Total PTGI-SF and domains	Correlation coefficient with total Benefit Finding Questionnaire
Appreciation of life	0.298*
New possibilities in life	0.434*
Relationship with others	0.475*
Spiritual development	0.367*
Personal strength	0.541*
Total PTGI-SF score	0.622*

*p < 0.001.

#### 3.2.4 Discriminant validity of the Chinese version of the PTGI-SF

As for discriminant validity of the Chinese version of the PTGI-SF, the findings are presented in Table 6. Pearson’s correlation coefficients between PTGI-SF domains and the HADS Depression subscale ranged from −0.278 to −0.053, while correlations with the HADS Anxiety subscale ranged from −0.056 to 0.102. The correlation coefficient between the total PTGI-SF and the HADS Depression and HADS Anxiety subscales were −0.178 and 0.028, respectively. These findings highlighted good discriminant validity of the Chinese version of the PTGI-SF (as all correlation coefficients < 0.3).

**Table 6:** Discriminant validity of the Chinese version of the Posttraumatic Growth Inventory-Short Form (comparison with the Hospital Anxiety and Depression Scale).

PTGI-SF domains and total score	HADS (Depression)	*p*-value
Appreciation of life	−0.063	0.152
New possibilities in life	−0.131	0.003*
Relationship with others	−0.156	< 0.001*
Spiritual development	−0.043	0.335
Personal strength	−0.278	< 0.001*
Total PTGI-SF score	−0.178	< 0.001*

PTGI-SF domains and total score	HADS (Anxiety)	*p*-value
Appreciation of life	0.022	0.619
New possibilities in life	−0.024	0.580
Relationship with others	0.021	0.636
Spiritual development	0.102	0.030*
Personal strength	−0.056	0.202
Total PTGI-SF score	0.028	0.526

^*^Statistical significance at *p* < 0.05.

#### 3.2.5 Exploratory factor analysis of the Chinese version of the PTGI-SF

The exploratory factor analysis findings of the Chinese version of the PTGI-SF are summarized in Table 7. Prior to extraction, data factorability was confirmed by a Kaiser–Meyer–Olkin measure of sampling adequacy of 0.755 and a statistically significant Bartlett’s test of sphericity (*p* < 0.001). Exploratory factor analysis of the PTGI-SF yielded three factors, with the initial factor showing an eigenvalue of 5.770 and the three factors together accounting for 57.70% of the total variance. Under Varimax orthogonal rotation, the factor loadings of all the items ranged from 0.525 to 0.815. Items 1 to 3 were designated to factor 1; items 4, 6, and 8 were designated to factor 2; and items 5, 7, 9, and 10 were designated to factor 3. The attempt to use maximum likelihood as the extraction method to extract four and five factors from the Chinese version of the PTGI-SF failed.

**Table 7:** Exploratory factor analysis of the Chinese version of the Posttraumatic Growth Inventory-Short Form using principal component analysis.

Item	Factor 1	Factor 2	Factor 3
1			0.637
2			0.590
3			0.757
4		0.771	
5	0.525		
6		0.773	
7	0.815		
8		0.560	
9	0.808		
10	0.579	0.533	
Eigenvalue	3.396	1.250	1.124
Total eigenvalue			5.770
Variance			57.70

Kaiser-Meyer-Olkin measure of sampling adequacy = 0.775, Barlett’s test of sphericity: Chi square = 265.69, *p* < 0.001.

#### 3.2.6 Confirmatory factor analysis of the Chinese version of the PTGI-SF

Table 8 presents the model fit indices for all three CFA models compared in this study. Model 1 is a unidimensional (one-factor) baseline model; Model 2 is the three-factor structure suggested by the EFA; and Model 3 is a modified unidimensional model with correlated residuals. Only Model 3 met the minimum threshold for acceptable fit and was therefore adopted. Table 9 reports the modification indices derived from Model 1, while Figure 1 illustrates the factorial structure of Model 3, displaying standardized factor loadings and the relevant correlated residuals.

**Table 8:** Confirmatory factor analysis of the Chinese version of the Posttraumatic Growth Inventory-Short Form (PTGI-SF).

Model	Model 1 One-factor baseline	Model 2 Three-factor EFA	Model 3 One-factor + correlated residuals
χ2/df	12.78	8.34	3.44
*P*-value	<0.001	<0.001	<0.001
CFI	0.872	0.927	0.978
TLI	0.835	0.897	0.966
RMSEA	0.171	0.135	0.078
SRMR	0.135	0.098	0.073

**Table 9:** Modification indices from Model 1 (One-factor baseline).

Variable_1	Operator	Variable_2	MI	EPC	Standardized_EPC
PTGI_1	~~	PTGI_2	37.490	0.330	0.462
PTGI_2	~~	PTGI_3	36.130	0.351	0.456
PTGI_1	~~	PTGI_3	15.750	0.256	0.316
PTGI_4	~~	PTGI_6	72.120	0.388	0.893
PTGI_7	~~	PTGI_9	194.070	0.634	2.375
PTGI_6	~~	PTGI_10	23.580	0.229	0.509

MI, Modification Index, signifying the magnitude of connection not accounted for by the model.

~ ~ = “related to”.

EPC, Expected Parameter Change, indicates the size of the connection.

Standardized_EPC, EPC put on a standard scale.

**Figure 1:**
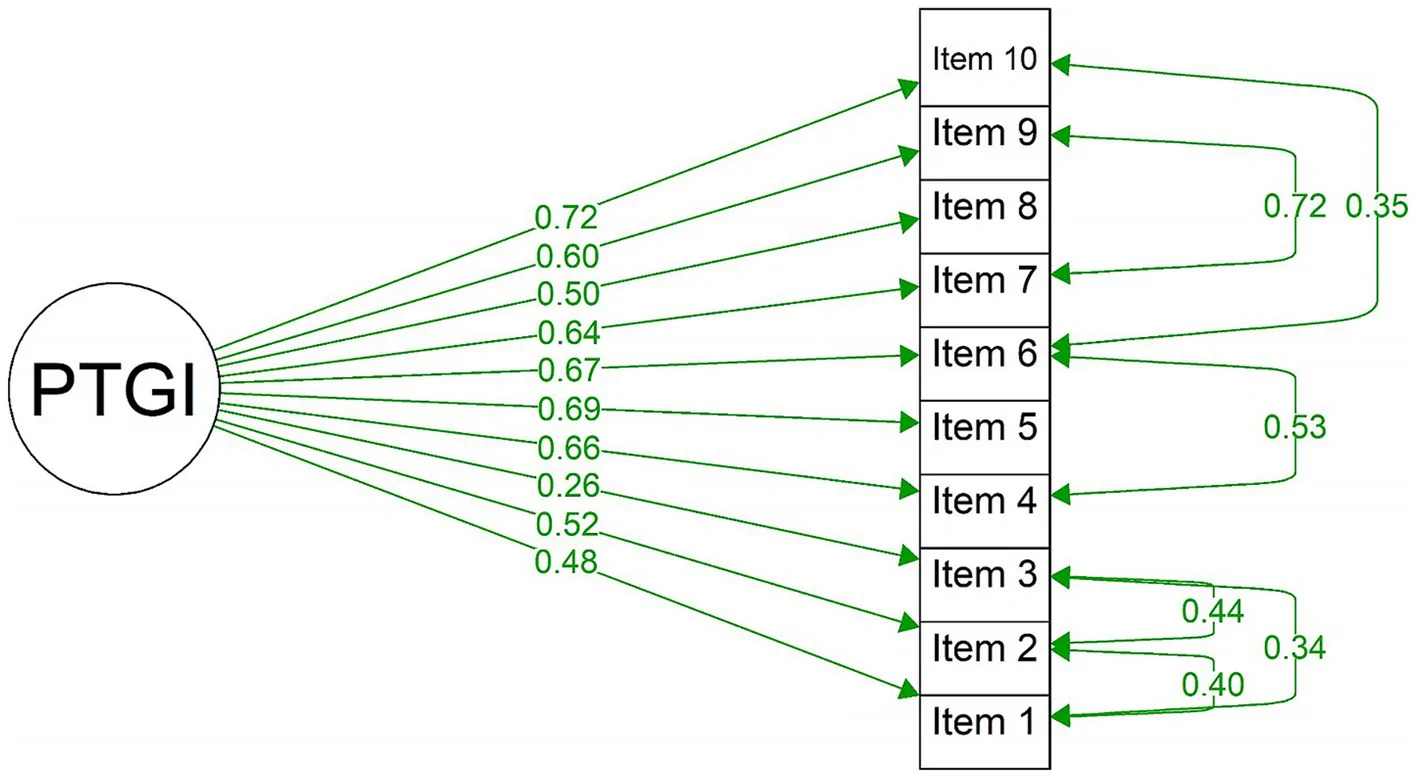
Unidimensional structure of PTGI-SF-C with standardized factor loadings and correlated residuals (Model 3).

#### 3.2.7 Psychometric evaluation of the items

The Rasch PCM revealed that the six-point ordinal scale of 0–5 for PTG-SF-C produced disordered category thresholds for all 10 items (Figure 2). Although the intended response sequence and structure from the original scale appear did not function as expected for participants in this study, the 10-item scale fit the Rasch model well. Infit MNSQ ranged from 0.72 to 1.39, and outfit MNSQ ranged from 0.64 to 1.27. Figure 3 demonstrates that all items are within the acceptable range of 0.50–1.50 for both fit measures.

**Figure 2:**
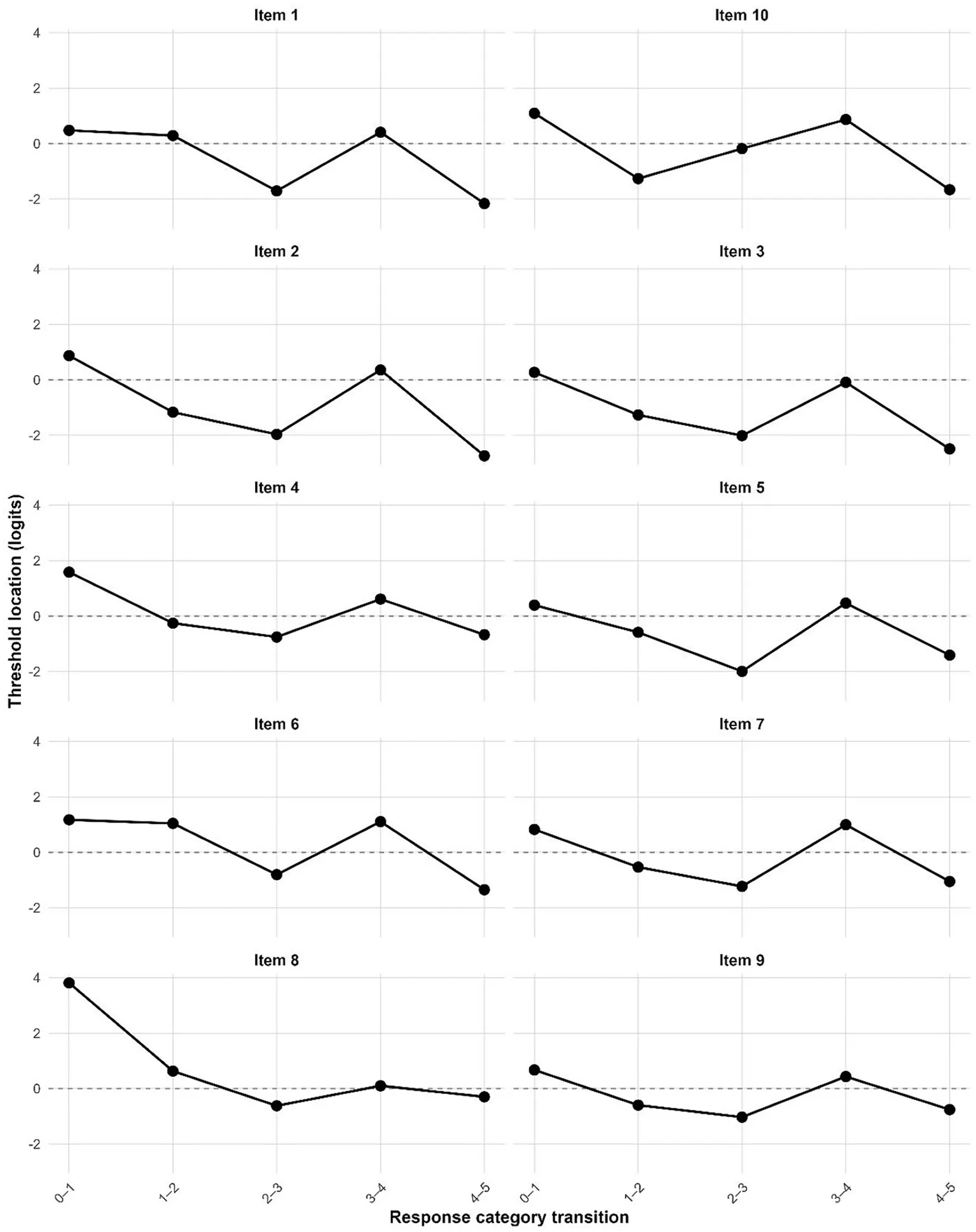
Category threshold across PTG-SF-C items. Non-monotonic threshold sequences indicate disordered functioning of the 0–5 response categories.

**Figure 3:**
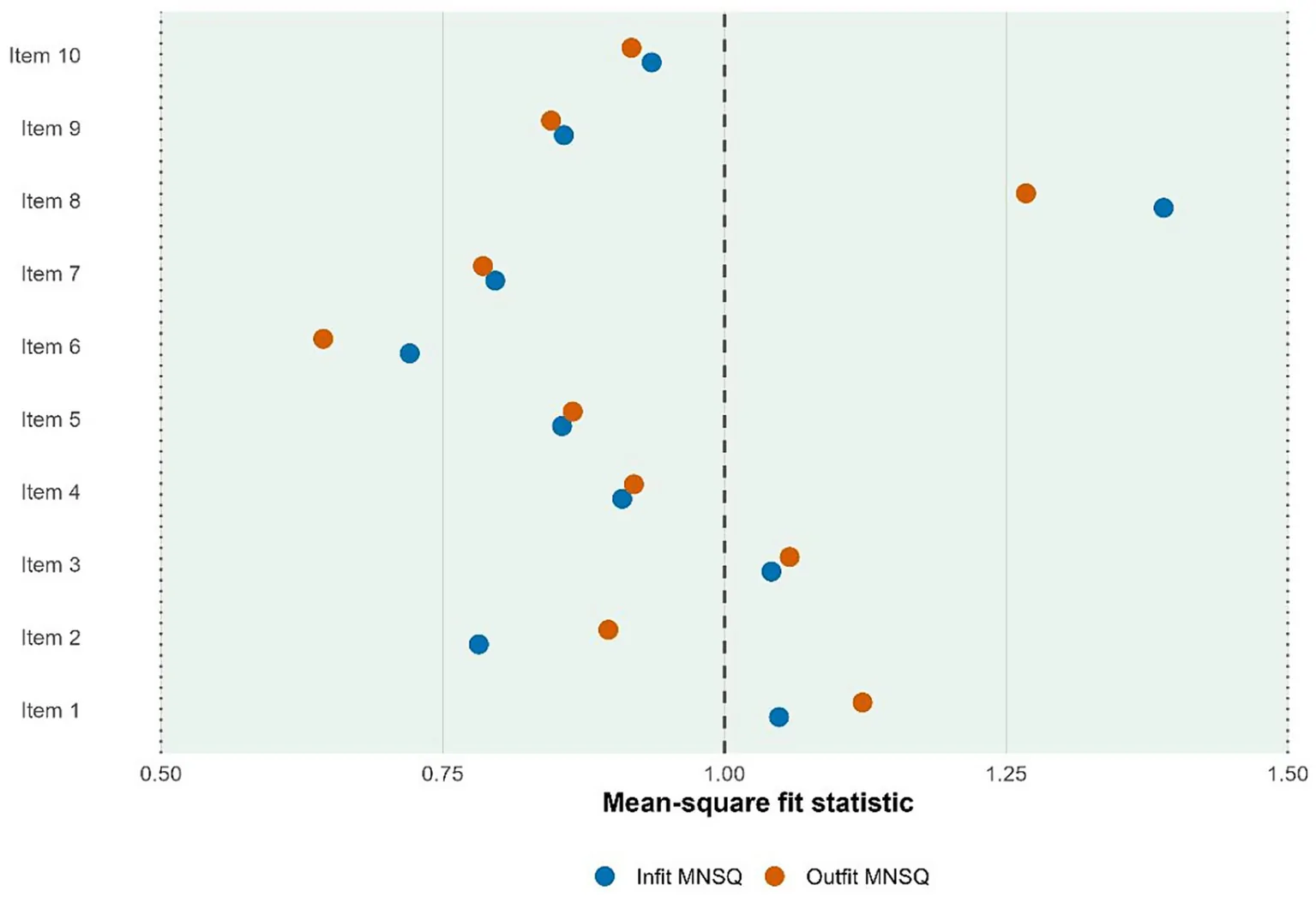
Rasch item fit of the Chinese PTGI-SF. Shaded region: Acceptable MNSQ range (0.5–1.5). Infit: Predictable responses given person ability. Outfit: Outlier sensitivity (e.g., guessing, bubbling the wrong circle, etc).

Additionally, person separation reliability and the separation index were 0.79 and 1.95, respectively, corresponding to approximately 2.93 distinguishable strata. These results suggest that the PTGI-SF-C could reliably identify about three distinct strata of individuals. The items are also matched to the latent trait of PTG, with the mean person measure standing at −0.31 logits, signifying close targeting between the scale and the respondents. Figure 4, meanwhile, illustrates that the PTG-SF-C measures PTG most accurately for lower-to-middle categories, where the curve of most items peaked. Differential item functioning (DIF) supports that each item of the scale shows no significant cultural or social bias attributable to gender. Figure 5 shows that items are in comparable locations, with the largest difference between males and females for Item 8 (−0.41 logits). Although the Andersen likelihood-ratio test [χ^2^(49) = 72.59, *p* < 0.05] showed an overall non-invariance by gender, no items had significant threshold parameters remaining after Benjamini-Hochberg correction.

**Figure 4:**
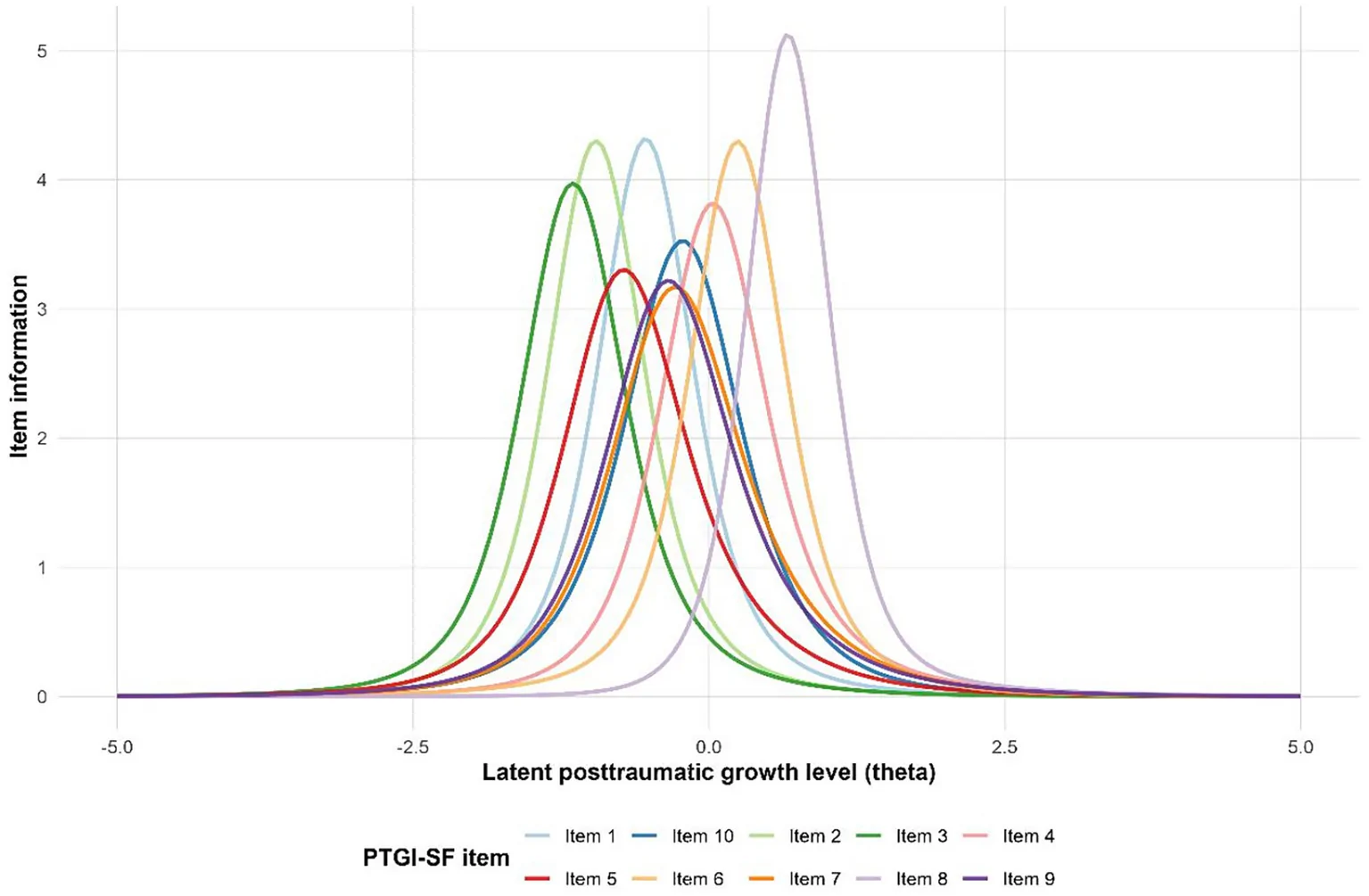
Rasch PCM item information functions. Higher curves indicate greater measurement precision at the corresponding level of posttraumatic growth.

**Figure 5:**
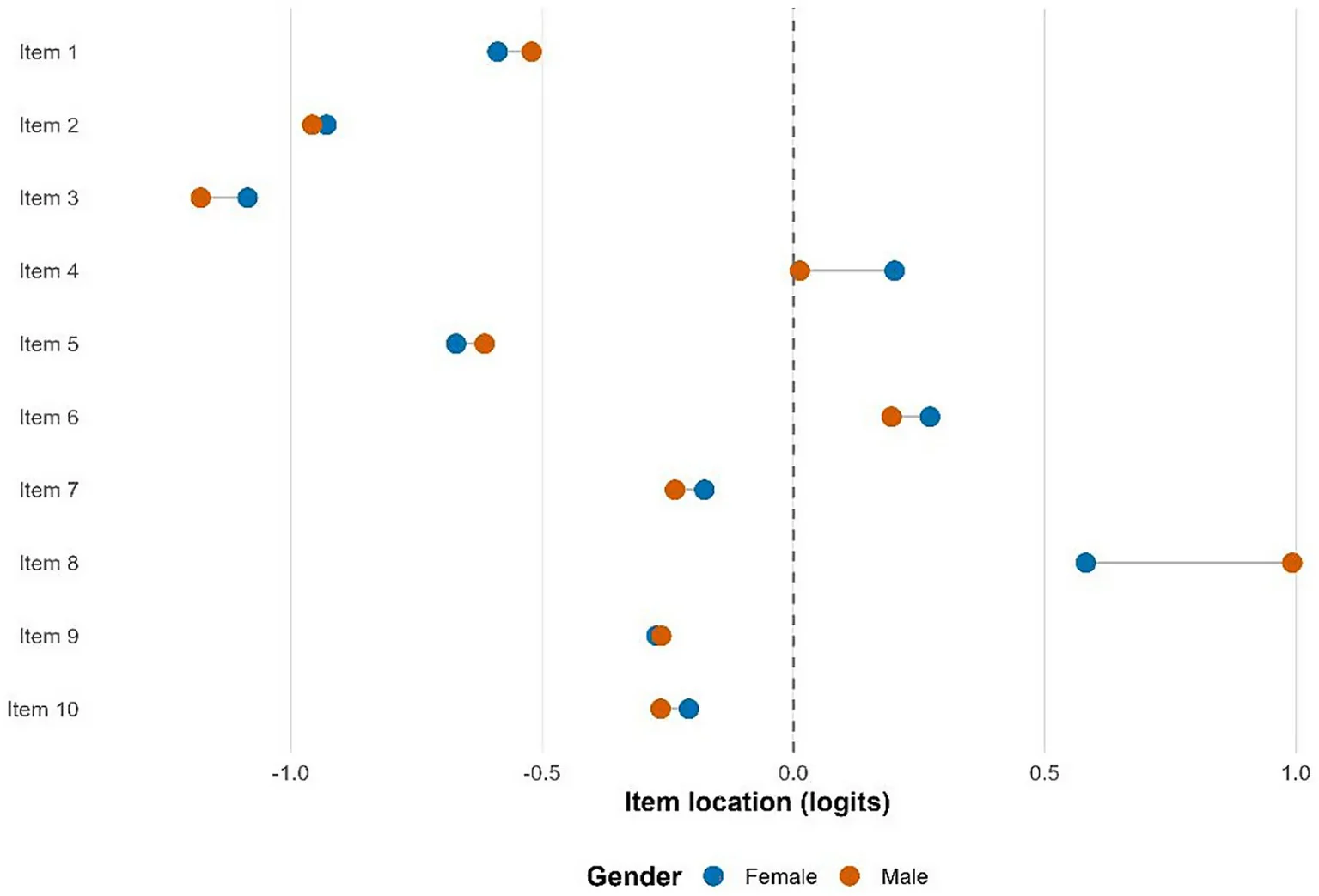
Differential item functioning (DIF) by Gender. Comparison of Rasch item locations between gender groups; inferential DIF is evaluated using Wald/LR test.

## 4 Discussion

The translation, adaptation, and validation of the Chinese version of the PTGI-SF among cancer patients in China confirmed that it had good psychometric properties. Regarding the reliability of the Chinese version of the PTGI-SF, the instrument had comparable internal consistency (Cronbach’s α = 0.89) with that of the original English version of the PTGI-SF (Cronbach’s α = 0.89). In comparison with the other translated versions of the PTGI-SF, the Chinese version of the PTGI-SF also had comparable internal consistency with the Spanish (Cronbach’s α = 0.83) (Castro et al., 2015), Portuguese (Cronbach’s α = 0.88) (Lamela et al., 2014), Persian (Cronbach’s α = 0.86) (Amiri et al., 2020), Urdu (Cronbach’s α = 0.88) (Naeem and Anila, 2019) and Malay versions of the PTGI-SF (Cronbach’s α = 0.89) (Leong Abdullah et al., 2017). The Arabic version of the PTGI-SF has slightly better internal consistency (McDonald’s omega = 0.95) (Fekih-Romdhane et al., 2024) compared with that of the Chinese version of the PTGI-SF (McDonald’s omega = 0.88). In terms of test–retest reliability, the Chinese version of the PTGI-SF also demonstrated comparable ICC (0.79) to that of the Malay version of the PTGI-SF (ICC = 0.75). Hence, the Chinese version of the PTGI-SF exhibited reasonable reliability with good internal consistency and test–retest reliability.

In the context of the translation of the Chinese version of the PTGI-SF from the original English version, the forward and back-translation of the Chinese version of the instrument, as well as the process of attaining and assessing content validity with a panel of content experts and face validity with a pilot study involving 30 cancer patients, followed the International Test Commission (ITC) Guidelines for Translating and Adapting Tests (Second Edition) (International Test Commission, 2017). The excellent I-CVI, S-CVI/Ave, and S-CVI/UA scores of the Chinese version of the PTGI-SF indicated that the instrument had good content validity (Table 1). While the excellent I-FVI, S-FVI/Ave, and S-FVI/UA scores of the Chinese version of the PTGI-SF denoted that the instrument had good face validity (Table 2) and did not require any further amendments by the expert panel.

Pearson’s correlation coefficients between the total score of the Chinese BFS and both the total score and several domains of the Chinese PTGI-SF exceeded 0.50, demonstrating good convergent validity (Chin and Yao, 2014). On the contrary, the Pearson’s correlation coefficient between all the domains of the Chinese version of the PTGI-SF and its total score, and the total score of the Chinese version of the HADS, were lower than 0.3, which demonstrated that the Chinese version of the PTGI-SF had good discriminant validity (Henseler et al., 2015).

However, the EFA of the Chinese version of the PTGI-SF demonstrated that the instrument had only three factors, deviating from the expected unidimensional structure of the scale. This is not surprising given the conceptual relatedness of the items, which may reflect clustering rather than a distinct underlying construct. Consequently, the following CFA compared three factorial structures: a one-factor baseline (Model 1), a three-factor structure suggested by the EFA (Model 2), and a one-factor model with theoretically and empirically supported correlated residuals (Model 3). Model 3 showed the best and most acceptable overall fit (Table 8), supporting the scale’s unidimensional structure with local dependencies between items (Figure 1). Cann et al. (2010) reported that the original English version of the PTGI-SF had five factors, and a good substitute for PTGI to assess the global indicator of PTG was mentioned; however, it was also mentioned that the PTGI-SF is not a good measure of the different domains of PTG, pinpointing the possibility of one-dimensionality of the PTGI-SF. In addition, the Arabic version of the PTGI-SF also confirmed a one-factor model as the best-fitting model (Fekih-Romdhane et al., 2024).

Complementary item-level evidence was provided by the Rasch PCM analysis. All items demonstrated acceptable infit and outfit MNSQ values, indicating that their response patterns were broadly consistent with Rasch model expectations. Infit statistics suggested adequate item functioning among respondents whose PTG levels were targeted by those items, while acceptable outfit statistics indicated no substantial influence of unexpected responses occurring far from respondents’ estimated locations on the latent continuum. Collectively, these findings support retaining all 10 items.

The gender DIF analysis provided generally reassuring, though not conclusive, evidence regarding measurement invariance. Most item-location estimates were similar for both men and women, and none of the individual threshold parameters remained statistically significant after applying the Benjamini-Hochberg correction. However, the significant Andersen likelihood-ratio test suggested some overall non-invariance of parameters across gender groups. Therefore, these findings should not be interpreted as demonstrating complete gender invariance. Instead, while robust DIF could not identify specific individual threshold parameters after correcting for multiple testing, there is still evidence of some overall gender-related parameter invariance. Further investigation is needed with independent and preferably larger samples, particularly for Item 8, which exhibited the largest descriptive difference in item location between men and women.

A particularly important finding was the presence of disordered category thresholds across all items (Figure 2). In an optimally functioning ordinal scale, successive thresholds should steadily increase along the latent continuum, reflecting respondents’ ability to distinguish progressively higher response categories. The observed disordering therefore suggests that the six-point ordinal response categories of the original PTGI-SF may not represent clearly distinguishable levels of PTG in this sample. This interpretation is also consistent with the markedly uneven category use observed for several items, indicating that some intermediate categories may contribute limited discrimination.

Separately, the item functions showed that measurement precision was greatest within the lower-to-middle region of the PTG continuum and decreased toward its extremes. Thus, although the items generally demonstrated acceptable Rasch fit, the instrument appears more precise for respondents located within this region than for those at very high or very low levels of PTG.

Taken together, these findings suggest an important distinction between item functioning and response-category functioning. The individual PTGI-SF-C items generally conformed to Rasch model expectations, whereas the six-category response structure showed evidence of suboptimal category functioning. Future studies should therefore investigate whether collapsing adjacent categories into empirically derived three-, four-, or five-category structures improves threshold ordering and measurement efficiency without substantial information loss. Such rescoring should be evaluated independently before recommending any modification to the original response format.

There were a few limitations to consider in this study. First, the study participants may not be representative of the cancer population in China, as the distribution of employment status, types of cancer, and mode of treatment received differ. Second, the study sample was recruited only from one tertiary center for oncology treatment in China. Hence, these limitations may affect the generalizability of the study findings.

Despite these limitations, the Chinese version of the PTGI-SF was translated and adapted for use in the cancer population in China and Chinese-speaking nations, and the tool was documented to have good psychometric properties. Future intervention studies investigating the efficacy of psychosocial programs to enhance PTG among Chinese-speaking cancer patients can now utilize this instrument to prospectively assess changes in PTG over time. Similarly, prospective studies to measure the trajectory of PTG across time since diagnosis or cancer treatment among Chinese-speaking cancer patients could now commence.

## 5 Conclusion

This study successfully translated and adapted the Chinese version of the PTGI-SF for use in measuring the degree of PTG among Chinese-speaking cancer patients and confirmed that the tool has sufficient internal consistency and test–retest reliability, as well as face, content, convergent, and discriminant validity. However, EFA and CFA confirmed that the Chinese version of the PTGI-SF is unidimensional, with the possibility of a single factor, which differs from the original English version and some of the translated versions of the instrument. The Chinese version of the PTGI-SF is now ready for use in the Chinese-speaking cancer population in China, Taiwan, Hong Kong, and Macao.

## Data Availability

The raw data supporting the conclusions of this article will be made available by the authors, without undue reservation.
