# Supplementary material for: Psychometric properties of the Chinese version of the Posttraumatic Growth Inventory-Short Form among Chinese-speaking cancer patients
Source: Front Psychol. 2026 Sep 23;17:1838732. doi: 10.3389/fpsyg.2026.1838732 (PMC13642187; doi:10.3389/fpsyg.2026.1838732)
Supplement: Supplementary file 1 [file Data_Sheet_1.PDF]

**Supplementary material 1. The characteristics of the expert panel in this study**

| Expert panel | Post | Qualification | Occupation | Experience | Role in study |
|---|---|---|---|---|---|
| Expert panel 1 | Psychiatrist | MD | Senior professor in psychiatry at Second Affiliated Hospital of Henan Medical University | More than 20 years of research work in psychiatry and cross-cultural validation of questionnaires | Content validity |
| Expert panel 2 | Psychiatrist | MD | Senior professor in psychiatry at Second Affiliated Hospital of Henan Medical University | More than 10 years of research work in psychiatry and cross-cultural validation of questionnaires | Content validity |
| Expert panel 3 | Clinical psychologist | PhD in Clinical psychology | Lecturer at Second Affiliated Hospital of Henan Medical University | 5 years of research work in psycho-oncology and cross-cultural validation of questionnaires | Content validity |
| Expert panel 4 | psychologist | PhD in psychology | Senior lecturer at Second Affiliated Hospital of Henan Medical University | 5 years of research work in mental health and cross-cultural validation of questionnaires | Content validity |
| Expert panel 5 | oncologist | PhD in clinical oncology | Senior professor in the First Affiliated Hospital of Henan Medical University | More than 20 years of research work in cancer and cross-cultural validation of questionnaires | Content validity |
| Expert panel 6 | oncologist | PhD in clinical oncology | Senior medical lecturer in the First Affiliated | 5 years of research work in psycho-oncology and cross-cultural | Content validity |

| | | | | | |
|---|---|---|---|---|---|
| | | | Hospital of Henan Medical University | validation of questionnaires | |
